# Crick Wobble and Superwobble in Standard Genetic Code Evolution

**DOI:** 10.1007/s00239-020-09985-7

**Published:** 2021-01-07

**Authors:** Michael Yarus

**Affiliations:** Department of Molecular, Cellular and Developmental Biology, University of Colorado Boulder, Boulder, CO 80309-0347 USA

**Keywords:** Coding, Triplet, Codon, Anticodon, Superwobble

## Abstract

Wobble coding is inevitable during evolution of the Standard Genetic Code (SGC). It ultimately splits half of NN U/C/A/G coding boxes with different assignments. Further, it contributes to pervasive SGC order by reinforcing close spacing for identical SGC assignments. But wobble cannot appear too soon, or it will inhibit encoding and more decisively, obstruct evolution of full coding tables. However, these prior results assumed Crick wobble, NN U/C and NN A/G, read by a single adaptor RNA. Superwobble translates NN U/C/A/G codons, using one adaptor RNA with an unmodified 5′ anticodon U (appropriate to earliest coding) in modern mitochondria, plastids, and mycoplasma. Assuming the SGC was selected when evolving codes most resembled it, characteristics of the critical selection events can be calculated. For example, continuous superwobble infrequently evolves SGC-like coding tables. So, continuous superwobble is a very improbable origin hypothesis. In contrast, late-arising superwobble shares late Crick wobble’s frequent resemblance to SGC order. Thus late superwobble is possible, but yields SGC-like assignments less frequently than late Crick wobble. Ancient coding ambiguity, most simply, arose from Crick wobble alone. This is consistent with SGC assignments to NAN codons.

## Introduction

### Calculation of the Evolution of Individual Coding Tales

Information below comes from simulation of the process of SGC evolution (see ‘Methods’). An era of early triplet assignment, decay and capture of new triplets is followed to a finished code. Time elapses in passages, computer visits to an evolving genetic code table (Yarus 2020a), which are proportional to real-world time. During a passage initial assignments, decays, and mutational capture of new triplets occur with assigned probabilities. Repeated passages yield complete coding tables (with all 22 functions), full coding tables (with 64 triplets assigned), and even near-full codes that are also near completion, proximal to the SGC itself (Yarus 2020b). Variation of the rules and probabilities for codon assignment allows calculation of evolved SGC frequencies (see ‘Methods’). Such frequencies determine how many independent codes would have to be examined to find an SGC-like code (Yarus 2020b). One can thereby seek the most likely route to SGC-like codes. Here, Crick wobble (Crick 1966) and superwobble (Rogalski et al. 2008) are compared in this way.

### First-Hand Information on Code Evolution

The genetic code evolves. Many evolutionarily recent departures from the near-universal code are known (Jukes and Osawa 1993), though a minority of codon assignments have been seen to change. Often, universal stop codons are modified (Osawa and Jukes 1989). Limited observable change is understandable among a complex biota which must compete with other highly selected systems, so that code change is rare. Modern code evolution is therefore said to be “frozen” (Crick 1968), though it might be called chilled. Nonetheless, modern changes offer important information. Altered assignments define practical variations and thereby, indicate low barriers over which evolutionary revision might go. Such indicators have their limits: They are most informative about the current nucleoprotein-based code, because they occurred in the molecular context of the modern SGC. So modern coding offers the most explicit information only about terminal stages of coding evolution. This is consistent with repeated recoding of stop codons, whose definitive encoding must have been late, after domain separation (Burroughs and Aravind 2019). However, modern changes necessarily also reflect the logic of coding itself, offering indirect guidance about the course of the likely ancestral, RNA-based code. Accordingly, modern coding variations are retroevolutionary pointers, defining usable routes toward the SGC.

### Examples of Change: Reassigned Termination Codons

A protist parasite of insects, Blastocrithidia, has reassigned UAA, UAG and UGA, thus altering all its ‘universal’ stop codons (Záhonová et al. 2016). Apparently, UAA and UAG can be translated as both glutamine and stop, while UGA has become a tryptophan codon. Terminal mRNA structure may determine when ambiguous translation as stop rather than an amino acid occurs (Swart et al. 2016). Similar ambiguous stop translation is common today, seen even in metazoa, as for hundreds of Drosophila genes (Jungreis et al. 2011).

### Examples of Change: Reassigned Termination Codons and New Amino Acids

Eubacterial selenium-containing enzymes have active sites translated using the ‘universal’ UGA stop as a codon for selenocysteine (the 21st amino acid). Encoding requires a dedicated aminoacyl-tRNA and special translation factor (Zinoni et al. 1990). Similarly, the Archaeal methanogen Methanosarcina uses the ‘universal’ UAG stop codon to co-translationally insert pyrrolysine (the 22nd amino acid) using a dedicated aminoacyl-RNA synthetase and tRNA (Polycarpo et al. 2004).

### Examples of Change: Unassigned Amino Acid Codons

The Gram-positive bacterium Mycoplasma capricolum has no adaptor to translate ‘universal’ CGG arginine (Andachi et al. 1989).

### Examples of Change: Reassigned Amino Acid Codons

The eukaryotic yeast Candida translates cytoplasmic ‘universal’ CUG leucine codons as serine, using a tRNA^Ser^ mutated to pair with the leucine codon CUG (Santos et al. 2011). The altered tRNA is mostly charged with serine, but is also acylated with a small minority of leucine. Coding reassignment may depend on evolutionary pressure from changing DNA base composition (Jukes and Osawa 1993) and/or an intermediate ambiguous encoding (Schultz and Yarus 1994). Such ambiguity is documented for Candida (Santos et al. 2011) and Blastocrithidia (Záhonová et al. 2016).

### Examples of Change: Unassigned Amino Acid Codons and Termination Codons

The complete genome of bacterium E coli has been replaced with synthetic DNA, making no use of ‘universal’ UCA and UCG serine, and simultaneously removing ‘universal’ UAG stops. The resulting bacterium has three unused codons, as a result of 1.8 × 10^4^ genomic codon changes. This is particularly impressive, because no overt functional selection was applied. In minimal growth medium at 37 °C, the recoded cell is quite competent, doubling in 1.7 × the parental bacterium’s time (Fredens et al. 2019). Thus, partial codes, even when they do not meet a selected requirement, are viable and functional: that is, legitimate evolutionary intermediates. In fact, the altered *E. coli* code resembles a computed evolutionary intermediate with unassigned sense and stop codons (Yarus 2020b).

### Alternate Wobbles

Informative coding changes extend beyond assignments, including also changed coding machinery. RNA adaptors, like aminoacyl-tRNAs, can pair to and translate more than one template codon using alternative base pairing, first recognized and called wobble by Frances Crick (Crick 1966) shortly after the genetic code was defined (e.g., Nirenberg et al. 1963). Nucleotide modifications enable a variety of such pairs with third codon nucleotides in modern coding (Grosjean and Westhof 2016). However, if one accepts a limitation to unmodified nucleotides, whose universal modern use makes a strong argument for ancient presence in the code, primordial wobble would include pairing to NN U/C and NN A/G codons, based on Crick’s (Crick 1966) G:U and U:G wobble pairs. Here this is termed Crick wobble, though this naming neglects Crick’s inosine wobble, because inosine is a modified A (as in Bass and Weintraub 1988).

### Superwobbles

Yeast Saccharomyces mitochondria (Bonitz et al. 1980) and fungal Neurospora mitochondria (Heckman et al. 1980) have only one tRNA to translate unmixed family boxes; that is, with all four codons NN U/C/A/G assigned to a single amino acid. For example, all alanine GC U/C/A/G translation is carried out with a single tRNA, having an unmodified U at its anticodon wobble position. Sometimes called ‘superwobble’, the same wobble system appears in bacterial Mycoplasma (Andachi et al. 1989) and tobacco Nicotiana plastids (Rogalski et al. 2008).

The genetic mechanism has been extensively worked out in tobacco plastids (Alkatib et al. 2012). In plastids, superwobble always exists in unmixed family boxes. However, translation is inefficient with respect to pairs of Crick-wobbling tRNAs or Crick wobble for NN U/C and overlapping superwobble in addition (Rogalski et al. 2008). Superwobble would also be strikingly appropriate for primordial coding: Simpler adaptor sets are needed for coverage of 20 assigned functions (van der Gulik and Hoff 2011), suited for fewer expressed genes, and appropriate for reduced levels of gene products (Vernon et al. 2001). An emerging genetic code plausibly also required a simplified translation apparatus, expressing only a few functions, and initially might not demand exceptional amounts of product. There is also a more specific rationale for superwobble. Continuous Crick wobble evolution has intrinsic difficulty evolving full codes, with all triplets assigned (Yarus 2020a). Superwobble, which assigns four codons at once rather than one or two, might increase wobble assignments via greater rates, extents, or both.

## Results

### Late Crick Wobble

**Fig. 1:**
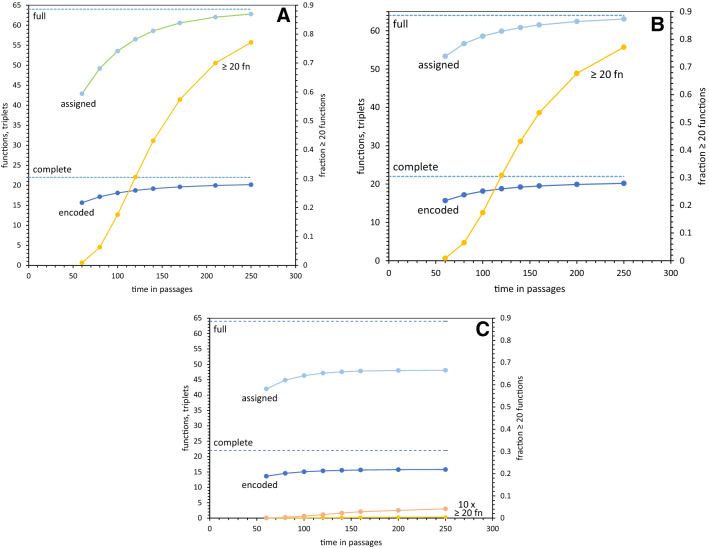
**a** Early progress of coding tables using late Crick wobble. Full coding is assignment of 64 codons. Complete coding is encoding of 22 functions—20 amino acids, initiation and termination. Assigned triplets have one of the 22 functions. Encoded functions have at least one triplet assigned to them. All data except ≥ 20 fn refer to the left ordinate; ≥ 20 fn is the fraction of coding tables with 20 or more functions encoded, and is plotted on the right. Data from 10^5^ evolutions. **b** Early progress of coding tables using late superwobble. Colors, axes and notation as in **a**. Data from 10^5^ evolutions. **c** Early progress of coding tables using continuous superwobble. Colors, axes and notation as in **a**. Data from 10^6^ evolutions

**Fig. 2:**
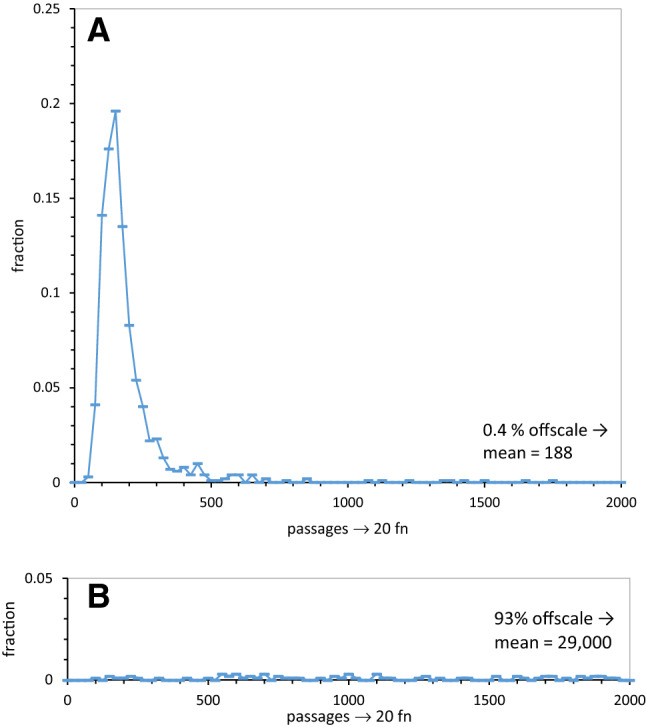
**a** Distribution of times to encode 20 pre-wobble functions uniquely, as precursor to late wobble. The fraction of 10^3^ evolutions that evolve to have 20 encoded functions at a specified time is plotted (in bins of 25 passages), using unique assignments. The fraction of the distribution taking longer than plotted times, and the distribution mean in passages is shown at lower right. **b** Distribution of times to encode 20 functions using continuous superwobble. The fraction of 10^3^ evolutions that evolve to have 20 encoded functions at a specified time (in bins of 25 passages), using continuous superwobble assignments. The fraction of the distribution taking longer than plotted times, and the distribution mean in passages is shown at lower right

**Fig. 3:**
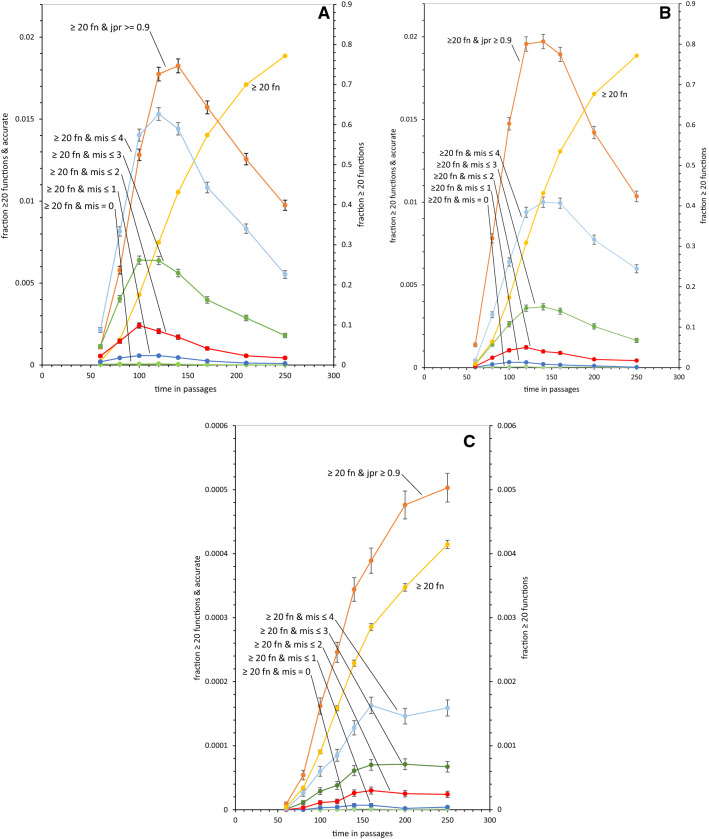
**a** Kinetics of SGC-like code evolution, late Crick wobble. All plots except ≥ 20 functions refer to the left ordinate; ≥ 20 fn refers to the right. “ ≥ 20 fn & jpr ≥ 0.9” indicates equal to or greater than 20 functions with joint progress equal or greater to 90% of the distance from randomized codes to the SGC. “ ≥ 20 fn & mis ≤ ..” indicates equal or greater than 20 functions and differences from SGC assignments less than or equal the cited number. Data from 10^5^ evolutions. All fractions have standard error bars, but these are not visible when they lie within data points. There were, at maximum, 8 × 10^–5^ codes with no misassignments. **b** Kinetics of SGC-like code evolution, late superwobble. As for **a**, except for late superwobble. There were, at maximum, 5 × 10^–5^ codes with no misassignments. **c** Kinetics of SGC-like code evolution, continuous superwobble. As for **a**, except with changed ordinate scales, and data from 10^6^ continuous superwobbling evolutions. There were, at maximum, 1 × 10^–6^ codes with no misassignments

The panels of Figs. 1, 2 and 3 compare average kinetics for coding table evolution following three different histories (Yarus 2020a). In Fig. 1a, late Crick wobble history is used: This implies that after an initial group of single-triplet assignments, translational mechanics required to make third position wobble specific and accurate (Moazed and Noller 1986; Ogle and Ramakrishnan 2005) evolve. Thereafter, Crick wobble is quickly adopted wherever possible in the nascent code (Fig. 1a). Such late adoption of wobble is the preferred path to SGC-like codes, because it easily evolves full coding tables, and also allows more frequent access to SGC-like (Yarus 2020a) codes. The alternative to late wobble is continuous wobble, where wobble exists throughout code evolution (Yarus 2020a).

For late Crick wobble, pyrimidine- and purine-ending codon groups, NN U/C and NN A/G, have the same assignment, but pyrimidine-ending codons can have different assignments from the purine-encoded triplets (Crick 1966). Such evolution (Yarus 2020a) easily approaches a full coding table (“assigned”, Fig. 1a) while simultaneously attaining coding capacity for 20 or more functions (“ ≥ 20 fn”, Fig. 1a), which becomes significant in a population after 60 passages. The average code evolves to a serviceable semifinal state, with sufficient codons left unassigned for later-evolving initiation and termination functions (Yarus 2020a), and perhaps a delayed amino acid (“encoded”, Fig. 1a).

### Superwobble Implementation

To emulate modern superwobble (Alkatib et al. 2012), Crick wobble and superwobble overlap in the event called “superwobble” here. That is, a newly assigned triplet can adopt Crick wobble, given that its wobble partner is free for such coding. If the other two triplets in its family box are also free, then it can expand to be translated by superwobble, creating identical assignments for NN U/C/A/G. But if either of the additional two triplets is already assigned, then coding stops at Crick wobble: NN U/C or NN A/G. To complete this assignment list, a triplet assigned a unique meaning during a pre-wobble era can also retain it, persisting as a single, non-wobbling codon (possibly with a differently assigned neighbor) into the later post-wobble era. When an assignment decays, its absence frees all triplets previously read for reassignment.

### Late Superwobble

Figure 1b presents mean results of superwobble implementation at the cited times, in passages. The results are much like Fig. 1a, for Crick wobble. However, more assignment in every use of superwobble, which can assign four codons at a time, appears in a greater number of codons occupied (“assigned”, Fig. 1b) just after 60 passages, when codes with near-complete coding capacity (“ ≥ 20 fn”) begin to appear. However, later behavior of Crick- and superwobble is similar, with full coding tables and near–complete coding appearing for both histories.

### Continuous Superwobble

Continuous superwobble, existing from the initiation of code evolution (Fig. 1c), is very different from late Crick and late superwobble, above. Marked differences appear in average codons occupied (“assigned”, Fig. 1c), in functions coded (“encoded”, Fig. 1c), and ultimately, in acquisition of near-full coding capacity (“ ≥ 20 fn”, Fig. 1c). All these indices of progress toward SGC capabilities are diminished or slowed.

Assignment of triplets does not approach full coding. Further, this average deficit stabilizes within the Figure. It is a property of the near-steady state—even given time, full assignment will not occur (Fig. 1c).

Capacity for near-complete encoding, ≥ 20 functions, accumulates very slowly. To make its kinetics visible, it is plotted at 1 × and 10 × its observed value in Fig. 1c. Whereas late Crick wobble and late superwobble population evolve to more than 77% near-complete coding in the early times shown in Fig. 1, continuous superwobble allows ≈ 200-fold less accumulated capacity.

Mean encoded functions reach about 15.8 of 22 amino acids/start/stop in Fig. 1c and this value is near-steady; it will not improve greatly. This is not just true of the mean; even the complete tables at the upper tail of the distribution are quite rare at ≈ 1 in 10^6^.

### The Difficulty with Continuous Superwobble

Figure 2 shows why the continuous wobble deficiency exists. It plots the fraction of coding tables that became capable of encoding 20 functions in bins of 25 passages, out to times of 2000 passages. Figure 2a is the relevant plot for any late wobble history, either Crick- or superwobble. It shows the acquisition of near-complete coding capacity during the early period of non-wobbling common to either late-wobbling scheme. Notably, near-complete coding occurs at a sharply defined early time. Late wobble evolution therefore quickly acquires, and virtually always confers, near-full coding capacity.

In contrast, Fig. 2b, for continuous superwobble, shows that coding capacity is delayed, and its average acquisition is at far later times than that for late wobble. Therefore, the probability that code evolution will reach this goal is small, at times when late wobble has already established near-full coding capacity.

### Coding Capacity and SGC-Like Assignments Together

One can evolve capacity to encode all functions and still not be SGC-like, if assignments differ from the standard code. Therefore, to evaluate an evolutionary history one wants to know how often a scheme yields coding capacity and SGC-like assignments together. These data are in Fig. 3, for the same range of early times as in Figs. 1 and 2.

### Coding Capacity with Accurate Assignment During Late Crick Wobble

Figure 3a shows joint competence for late Crick wobble alone, thus overlapping previously presented data (Yarus 2020a, 2020b). The plot for mean coding capacity, ≥ 20 functions, from Fig. 1a is shown again to facilitate comparisons. Coding capacity accompanied by accurate assignments is plotted in six accompanying curves.

Five of these plots result from counting assignments that differ from the SGC. Thus the data labeled “ ≥ 20 fn & mis ≤ 4” is the fraction of coding tables that encode 20 or more functions with less than or equal to 4 misassignments by comparison to the SGC. At their optimum, these capable ≤ 4 misassignment codes comprise 0.0153 or 1.53% of all late-wobbling coding tables.

Notably, these data also descend to small, but finite values for “ ≥ 20 fn & mis = 0”, which represent elevated coding capacity with no differences at all from SGC codon assignments. These are rarer, as expected: 0.00008 or 0.008% of late-wobbling evolutions.

Finally, evolution of joint competence is evaluated for encoding of 20 or more functions along with previous indices of SGC-like order (Yarus 2020a). Rather than counting misassignments, order is measured via SGC-like spacing in identical assignments, close spacing of assignments with similar side chain chemistry (Woese 1965; Mathew and Luthey-Schulten 2008), and mutational distance from the SGC. To be accounted “close”, a coding table must be ≥ 90% the distance from random codes to the SGC, for all three progress values (termed “jpr (joint progress) ≥ 0.9”). This is the topmost plot, showing “ ≥ 20 fn & jpr ≥ 0.9” achieved in 0.0183 or 1.83% of all evolutions.

Notably, coding capacity with accurate assignments and coding capacity with SGC-like order have overlapping maxima at an early time, as previously pointed out (Yarus 2020b). Both capacity-plus-order criteria then decrease at later times. So, there is an early optimal era during which late Crick-wobbling coding tables most resemble the SGC itself, using indices of both SGC-like order and codon assignment (Yarus 2020b).

### Distribution Fitness for Late Crick Wobble

At the 120 passage maximum, coding capacity with SGC-like order exists in ≈ 1.8% of code evolutions, ≈ 1.5% have coding capacity with ≤ 4 differences from the SGC, 0.64% capacity and ≤ 3 differences—down to 0.008% with SGC assignments only. This defines a varied population that can be tested to select a code. The property called distribution fitness (Yarus 2020a) for late Crick wobble is established; very close relatives of the SGC are available. This is significant in itself, but also, such data from times across the optimum argue that if the SGC arose during the era when evolving codes most resemble the SGC, then a nascent SGC could have resembled codes evolved here (see 'Discussion').

### Coding Capacity with Accurate Assignment During Late Superwobble

Figure 3b shows data paralleling Fig. 3a, but for late superwobble rather than late Crick wobble. As for Figs. 1a, b and 3 data are somewhat similar. Crick and superwobble data are plotted against the same set of ordinates, and accompanied by their similar ≥ 20 function plots, to facilitate such comparison.

Notably, while progress values (order) and coding capacity are similar for the two histories, assignment accuracy differs. Superwobble reproducibly yields less accurate assignments. This difference is only slightly varied among 0, ≤ 1, ≤ 2, ≤ 3 or ≤ 4 misassignments, so Crick wobble evolution yields, on average, an optimum of ≈ 1.7-fold more frequent SGC-like assignment than superwobble at all levels of accuracy. In particular, this applies to the no-error, mis = 0 assignment identity class—1.6-fold more frequent for Crick wobble than for superwobble.

### Coding Capacity with Accurate Assignment During Continuous Superwobble

Figure 3c parallels the first panels of Fig. 3 for late Crick and superwobble, but instead, is computed for continuous superwobble as coding history. To appreciate the differences, note that ordinates in Fig. 3c are smaller than the rest of Fig. 3; smaller by large, order-of-magnitude factors. Continuous superwobble radically reduces both coding capacity (as observed in Figs. 1c and 2b), and the resulting abundance of capable, accurately assigned coding tables. This deficit appears in accuracy assessed as both overall order (joint progress; “ ≥ 20 fn & jpr ≥ 0.9”, Fig. 3c) or literal assignment accuracy (“ ≥ 20 fn & mis…”,  Fig. 3c).

An optimal time does not exist for continuous superwobble in the same sense as for late Crick and late superwobble histories (Fig. 3c). Varying amounts of wobble when it is instituted at different times create the optimum for late Crick wobble (Yarus 2020b) and late superwobble (Fig. 3b). Continuous superwobble does not share a comparable effect. But again, constant superwobble’s net effect is similar when measured at different levels of assignment accuracy (cf. Figure 3a, c). So its effect can be summarized: continuous superwobble depresses the evolution of combined coding capacity and assignment accuracy, with respect to best late Crick wobble, by ≅ 100-fold.

## Discussion

Here late Crick wobble, late superwobble and continuous superwobble are compared (see ‘Methods’), quantifying their effects on evolving coding tables. These effects are assessed throughout an early era when coding approaches the ≥ 20 function capacity required for an SGC (Fig. 1). The emphasis is: Does superwobble (NN U/C/A/G translation by one adaptor) aid SGC-like evolution?

### Previous Implications are Strengthened

Comparison of continuous and late superwobble parallels prior work (Yarus 2020a), where continuous Crick wobble and late Crick wobble were compared. Late Crick wobble previously appeared superior, because it both allowed fuller coding, and created more frequent access to the SGC. Here again, late superwobble allows fuller coding (Fig. 1b, c) than does continuous superwobble, and also much more frequent access to full, accurate, SGC-like assignments (Figs. 2b and 3b, c). Moreover, while the greater span of superwobble coding ambiguity can slightly increase early assignment (Fig. 1a, b), it does not correct continuous wobble’s deficit in near-steady-state assignments (Fig. 1c). Finally, though late superwobble shares late Crick wobble’s approach to full and complete coding (Fig. 1a, b), its quadruple assignments do not increase overall code order (Fig. 3a, b). Continuous superwobble actually decreases codon assignment accuracy, measured as SGC-like assignments in near-complete codes (Fig. 3a–c). One’s impression is: wobble helps structure the code (Yarus 2020a), but too much such help is counterproductive. The best wobble is the least that is sufficient. Late wobble is better than continuous wobble, Crick wobble is better than superwobble.

Notably, late superwobble shares late Crick wobble’s early maximum (≈ 120 passages), when both overall code order and accurate assignments appear maximally and nearly simultaneously (Yarus 2020b), Fig. 3a, b). Early selection of an SGC-like code, when it is most prevalent, is strengthened by these data, showing that such an optimum exists for different late wobble systems.

### Late Crick Wobble

Continuous Crick wobble only partially assigned the 64 triplets, yielding coding tables lacking ≈ 20 triplet assignments (Yarus 2020a). In addition, continuous Crick wobble had three– to fourfold less likely access to the SGC. “Less likely access” is given its evolutionary meaning by the abundance equation:

$$E = {\raise0.7ex\hbox{${\ln 2}$} \!\mathord{\left/ {\vphantom {{\ln 2} {P_{{{\text{event}}}} }}}\right.\kern-\nulldelimiterspace} \!\lower0.7ex\hbox{${P_{{{\text{event}}}} }$}}$$

where abundance of a desired code, *P*_event_ determines *E*, the number of independent codes that must be examined to find, with probability = 0.5, the rare desired one (Yarus 2020b; see ‘Methods’). So, continuous Crick wobble required a three– to fourfold larger population than late Crick wobble to find capable, SGC-like codes. Here, late Crick wobble again fills coding tables, leaving appropriate room for known late coding (Fig. 1a) and arising via an abundant, quickly appearing group of intermediates (Fig. 2a). Moreover, late Crick-wobbling codes are accessible, meaning even odds that accurate examples are found among 45 independently formed codes (≤ 4 misassignments; Fig. 3a) to 8700 independent codes (if no misassignments must occur; Fig. 3a).

### Late Superwobble

Late superwobble shares with late Crick wobble near-full codes that are near-complete (Fig. 1a, b). This is because the pre-late-wobble era quickly provides both histories with elevated coding capacity (Fig. 2a). As a result, late Crick wobble and late superwobble have very similar access to combined coding capacity and SGC-like order (“ ≥ 20 fn & jpr ≥ 0.9”, Fig. 3a, b). In addition, late Crick wobble and late superwobble are somewhat similar if coding capacity and assignment accuracy are reckoned (“ ≥ 20 fn & mis…”,  Fig. 3a, b). However, there is a quantitative difference: Late superwobble is reproducibly less effective, evolving maximally fit codes at 0.58 the frequency of late Crick wobble (Fig. 3a, b).

### Continuous Superwobble

Discussion of superwobble ends with the history least likely to contribute to the SGC. Late superwobble and continuous superwobble differ greatly. Continuous superwobble, throughout code evolution, obstructs the evolution of code order (Fig. 3b, c), measured by joint progress (jpr: See ‘Methods’). Ordered coding capacity (with joint order ≥ 90% of SGC levels) is about 100-fold less frequent for continuous superwobble than for late Crick wobble (Fig. 3a, c) at the time when late superwobble has maximum SGC resemblance (≈ 120 passages; Fig. 1a). The intuitive expectation (see 'Introduction') that superwobble’s simultaneous assignments might help compose full coding tables is briefly realized (Fig. 1a, c), but only early, just after near-complete codes begin to appear. Later, continuous superwobble leaves an average of 16 triplets unassigned and does not usually complete coding, leaving an average of 6.2 functions unencoded. These deficiencies are due to completion complications (Yarus 2020a; Fig. 2b), kinetic difficulties in making late assignments to complete a code. Such complications are particularly severe for continuous superwobble (Fig. 2b). What is true of averages is also true of the upper tails of these distributions: At maximum, less than 1 in 10^6^ continuously superwobbling codes are complete.

Low assignment accuracy is confirmed by misassignment counts (Fig. 3c); continuous superwobble is 100-fold less able to evolve the combination of coding capacity with accurate assignments, at any level of misassignment.

### Selection of the SGC

Thus, suppose that the SGC was selected when populations resembled it. Then, at any time within the era when nascent codes most resemble the near-universal genetic code—and whatever assignment accuracy was required to meet that selection—and whether SGC-like order or literal assignment accuracy or both were selected, it is very unlikely that coding tables evolving with continuous superwobble would have been chosen. This is especially likely with contemporaneous late Crick wobble, which supplies codes of equivalent quality 100 times more frequently.

### Appraisal of Wobble and Superwobble

Primordial late superwobble cannot be ruled out. Throughout the era when late-wobbling codes most resemble the SGC, late superwobble coding is well-ordered, but moderately disfavored because it makes fewer SGC-like assignments. In contrast, continuous superwobble’s distribution fitness (Yarus 2020a) is abysmal. It seems fair to summarize: No meaningful advantage has been detected for superwobble; instead, it orders the coding table well, but offers moderate to severe disadvantages in assignment accuracy. The simplest inference is that late Crick wobble can account for SGC evolution. Accordingly, superwobble seems an adaptation for simplifying modern nucleoprotein coding (Vernon et al. 2001).

### Comparison with the SGC: The NAN Column

**Fig. 4:**
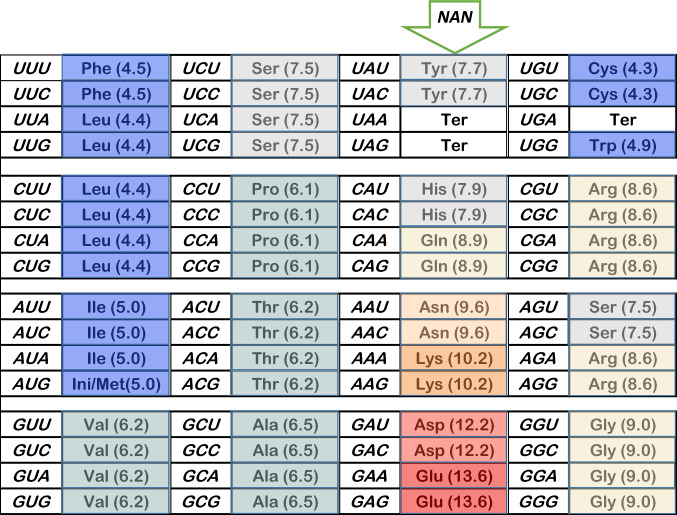
The SGC with color-coding for polar requirement. The Standard Genetic Code, with polar requirements for amino acids (Woese 1965; Mathew and Luthey-Schulten 2008) in parentheses beside assignment abbreviations. The color scale runs, in 1 pH unit blocs (e.g., 6.01–7.00), from blue (very hydrophobic) through light blue, gray, beige, yellow, light red, to dark red (very polar). Color meaning can be deduced from parenthetical numbers. The NAN column (codons with central A, N is any other nucleotide) is marked for discussion

In the following, it is assumed that the early code approximates the modern one. That is, ancient RNA-based and modern nucleoprotein codes likely have continuity (Orgel 1968), because it would have been deleterious to extensively alter coding during emergence of a nucleoprotein SGC with essential peptides encoded. Put another way, the form of the transitional SGC was already “frozen” (Crick 1968). Figure 4 is the SGC, with triplets colored to visualize chemical character as polar requirement (Woese 1965; Mathew and Luthey-Schulten 2008). The NAN column (codons with a central A, flanked by any nucleotides) is marked for discussion. During NAN specification (Fig. 4), 8/22 encoded functions and 16/64 triplets were assigned, a substantial fraction of the complete code.

In the NAN column, the negative quantitative conclusion above, about superwobble, is supported by a positive qualitative argument: There seems to have been a considerable time when only Crick wobble assignments occurred in the nascent SGC.

### The NAN Column Suggests an Era of Chemical Decisions

Columns of assignments with similar properties have long been recognized as a principle that organizes early genetic coding (Massey 2006; Higgs 2009). However, NAN codons represent chemically varied amino acids (Fig. 4): aromatic, aliphatic, neutral polar, positive and negative side chains. Accordingly, NAN assignments make little sense by usual amino acid grouping criteria. NAN amino acids are both prominent in Miller discharge (Miller 1987) experiments (Asp, Glu) and also absent (e.g., His; Higgs and Pudritz 2009). Consensus primordial amino acid lists, consulting 60 (!) chemical criteria include them, and also do not (Trifonov 2004). They are mixed in assignment to the two classes of aminoacyl-tRNA synthetases (Wetzel 1995). NAN amino acids are synthesized in varied ways, products of different anabolic pathways (Wong 1981; Taylor and Coates 1989).

But NAN column assignment is unified by encoded side chain chemistry. As shown in Fig. 4, the NAN amino acids are not only grouped, but show a remarkable, consistent trend in polar requirement from a neutral top (UA U/C Tyr) to an exceedingly polar bottom (GA A/G Glu). Termination codons (UA A/G) are necessarily excepted from the polar requirement comparison, though they may stand in for a lost primordial residue (Yarus 2001). Moreover, NAN assignment was probably extended over time: Upper codons have chemically similar assignments alongside (Ser alongside Tyr, Gln alongside Arg, perhaps Ter). These are likely mutational captures, in which the central anticodon nucleotide changes and an adaptor is reassigned to a similar amino acid (Yarus 2020a). In contrast, the lower 4 assignments everywhere contrast chemically with their lateral neighbors. This strikingly isolated reddish lower polar peninsula was formed by first and third position mutation alone to encode more extreme polarities, perhaps too quickly for intervening second position change, as seen at the top of the NAN column.

Details aside, the NAN column suggests that ever more polar amino acids were assigned after first and third codon position mutation alone, in each case by assignment to an adaptor RNA using Crick wobble. For example, this pattern could be a fossil signifying ever larger peptide structures, which made stronger and stronger distinctions between hydrophobic interiors and polar exteriors, where similar amino acids are prominent today (Miller et al. 1987). NAN assignments suggest substantial SGC evolution using only Crick wobble assignments.

### Increased Bayesian Convergence

These data add to the credibility (Yarus et al. 2005; Yarus 2020a) of a late wobble route to the SGC. Not only does late wobble explain full coding, and make SGC access more probable (Fig. 1a, b, Yarus 2020a), but new assignment using late Crick wobble also rationalizes SGC coding bloc structure (Fig. 4).

## Methods

### Computation

Calculations were performed on a Dell XPS computer, with an Intel i9-8950HK CPU @ 2.9 GHz and 31.7 GB RAM, under 64-bit Windows 10 v. 1909. Code evolution software was written and run in console mode using the Lazarus Pascal IDE v1.8.4, then passed, using tab-delimited output files, to 32-bit Microsoft Excel 2016 for further analysis and graphic output. This arrangement allowed analysis of up to 10^6^ coding table evolutions. A copy of the ≈ 900 line Pascal source and its associated Excel file are available on request.

### Evolution Software

The code evolution program (Yarus 2020a) uses Mersenne-twister randomized numbers (with a changing seed) to choose a triplet from a standard coding array, then executes one and only one of the following randomly chosen events at the chosen triplet. Initiation (initial codon assignment) with probability *P*_init_ = 0.6; decay to unassigned status (for assigned codons) with probability *P*_decay_ = 0.04; mutational capture (capture of an unassigned codon by an assigned one for its existing, or for a related, amino acid) with probability *P*_mut_ = 0.04. If none of these stochastic events occurs, one passage is over, and the program proceeds to the next, randomly chosen, triplet. This protocol is equivalent to assigning first-order rate constants to initiation and decay, and a second-order rate constant to mutational capture, with a passage as the unit of time (Yarus 2020a).

### Mode of Evolution

Initiations are randomly chosen SGC assignments 90% of the time. This can be rationalized if crucial assignments were stereochemical (Yarus 2017). 10% random codon assignment (*P*_rand_ = 0.1) is near the upper limit for evolution of SGC-like coding (Yarus 2020a). Mutational capture uses the protocol previously called Coevo_PR (Yarus 2020a), in which assignments to a related codon are made to a metabolically related amino acid (Wong 1981; Di Giulio 1991), but preferring metabolic relations that also have related polar requirements (Woese 1965; Mathew and Luthey-Schulten 2008). This logic, with probability of coevolutionary assignment increasing linearly as polar requirement (chemical) difference decreases, is used for examples because it most frequently yields SGC-like codes (Freeland and Hurst 1998; Yarus 2020a).

### Superwobble Implementation

For simplicity, codons have only one adaptor. In this work, that tRNA-like molecule pairs uniquely (Yarus 2020a), to two Crick-defined codons (Crick 1966), or superwobbles to four codons varying at the third position (Bonitz et al. 1980; Andachi et al. 1989). When an assignment decays, the evolving coding table loses one, two or four assignments. Thus, assignment or loss of either one, two, or four codons with the same two initial nucleotides are the elementary coding events.

### Evolutionary Success: Joint Progress

Success of a particular evolutionary history is evaluated by counting, among codons that have been assigned, differences from the SGC. But an alternative is to measure code order by counting evolved codes that are ≥ 90% of the distance from mean randomized coding tables to the SGC. Joint progress (0 ≤ jpr ≤ 1) is that number or fraction, for three distance criteria simultaneously (Yarus 2020a): Spacing (mean mutational distance between identical assignments, per triplet pair), distance (mean mutational distance to identical SGC assignments, per triplet pair) and dPR (mean distance in polar requirement units to codons that differ by single mutations, per triplet pair).

### The Abundance Equation

By repeating the above-described coding table evolution, the abundance of rare SGC-like codes can be determined. For example, codes that utilize assignments that do not differ from the standard genetic code are of particular interest (Fig. 3). Observed abundances, *P*_event_, can be given a more intuitive evolutionary meaning by conversion into *E*, the mean number of independent codes that must be surveyed to observe the event (Yarus 2020b):

$$E = - \frac{{\ln \left( {1 - P_{{{\text{obs}}}} } \right)}}{{P_{{{\text{event}}}} }} = \frac{{\ln 2}}{{P_{{{\text{event}}}} }}$$

where *P*_obs_ is the fraction of rare events (*P*_event_ < ≈ 0.1) observed. The second equality is for *P*_obs_ = 0.5; ‘even odds’ of detection.
